# circIQCH sponges miR-145 to promote breast cancer progression by upregulating DNMT3A expression

**DOI:** 10.18632/aging.103746

**Published:** 2020-08-03

**Authors:** Yuehua Li, Baohong Jiang, Zhengxi He, Hongbo Zhu, Rongfang He, Shanji Fan, Xiaoping Wu, Liming Xie, Xiusheng He

**Affiliations:** 1 Department of Medical Oncology, The First Affiliated Hospital, University of South China, Hengyang 421001, Hunan Province, China; 2 Key Laboratory of Cancer Cellular and Molecular Pathology in Hunan Province, Cancer Research Institute, Hengyang Medical College, University of South China, Hengyang 421001, Hunan Province, China; 3 Department of Pharmacy, The First Affiliated Hospital, University of South China, Hengyang 421001, Hunan Province, China; 4 Department of Oncology, Xiangya Hospital, Central South University, Changsha 410008, Hunan Province, China; 5 Department of Pathology, The First Affiliated Hospital, University of South China, Hengyang 421001, Hunan Province, China; 6 Department of Breast and Thyroid Surgery, The First Affiliated Hospital, University of South China, Hengyang, 421001 Hunan Province, China

**Keywords:** circIQCH, circular RNAs, DNMT3A, competitive endogenous RNAs, breast cancer

## Abstract

As a unique type of RNA, circular RNAs (circRNAs) are important regulators of multiple biological processes in the progression of cancer. However, the potential role of most circRNAs in breast cancer lung metastasis is still unknown. In this study, we characterized and further investigated circIQCH (hsa_circ_0104345) by analyzing the circRNA microarray profiling in our previous study. circIQCH was upregulated in breast cancer tissues, especially in the metastatic sites. CCK-8, transwell, wound-healing and mouse xenograft assays were carried out to investigate the functions of circIQCH. Knockdown of circIQCH inhibited breast cancer cell proliferation and migration to lung. Moreover, luciferase reporter assays and RNA immunoprecipitation assays were performed to elucidate the underlying molecular mechanism of circIQCH. The results showed that circIQCH sponges miR-145 and promotes breast cancer progression by upregulating DNMT3A. In summary, our study demonstrated the pivotal role of circIQCH-miR-145-DNMT3A axis in breast cancer growth and metastasis via the mechanism of competing endogenous RNAs. Thus, circIQCH could be a potential therapeutic target for breast cancer.

## INTRODUCTION

Breast cancer is the most pervasive malignancy and the second leading cause of cancer-related deaths among women, according to the estimated cancer statistic in the world [1]. Despite the innovation and progress made in systematic treatment (surgery, chemotherapy, hormonal therapy, targeted therapy and etc.), some patients with breast cancer will still develop metastatic disease after surgery, especially the triple negative breast cancer [2–3]. Accounting for over 90% of the death cases, metastasis is the main reason for breast cancer mortality [4]. Patients suffering from breast cancer lung metastases have a poor prognosis with a median survival time of 21 months [5]. To some extent, metastatic breast cancer is unresectable and the treatment is limited to systematic treatment with unsatisfied efficacy [6, 7]. Thus, it is urgent to identify novel accurate biomarkers and develop new therapeutic targets for patients with breast cancer.

Recently, circular RNAs (circRNAs) have become a hotspot in the field of biomedicine and have been widely studied. As a novel type of endogenous noncoding RNAs (ncRNAs), circRNAs widely existed and expressed in mammalian cells with a cyclic ring structure [8]. With highly conversed sequences and stable structure, circRNAs formed by the back splicing of exons or introns without a head or a tail [9]. Being the mediators of a variety of biological process in the cell, circRNAs regulate the expression of key genes via multiple comprehensive mechanisms, including sponging microRNA (miRNA), binding proteins and encoding novel proteins [10]. Scientists have discovered that circRNAs are the vital regulators of the process of multiple diseases, including diabetes, Alzheimer's disease, heart failure, cancer and so on [11–14]. Thanks to the efforts of researchers, its special roles in the development and the progression of cancers were gradually uncovered [15]. One of the most famous and well-studied circRNAs, CDR1as promotes growth and metastasis of different tumors by sponging miR-7 [16–19]. circFBXW7 is downregulated in tumor tissues which can inhibit cell proliferation and metastasis in glioma and triple negative breast cancer by encoding a 21kDa novel protein FBXW7-185aa and blocking miR-197-3p [20–21]. However, the potential functions and the underlying molecular mechanism of the most circRNAs are still unclear.

In this study, we characterized a frequently upregulated novel circRNA hsa_circ_0104345 in metastatic breast cancer by reanalyzing the circRNA microarray profiling in our previous study. circIQCH was upregulated in breast cancer tissues, especially in the metastatic sites. Knockdown of circIQCH inhibited breast cancer cell proliferation and migration to lung. Luciferase reporter assays and RNA immunoprecipitation assays were performed to elucidate the underlying molecular mechanism of circIQCH. Briefly, our study demonstrated the pivotal role of circIQCH-miR-145-DNMT3A axis in breast cancer growth and metastasis via the mechanism of competing endogenous RNAs.

## RESULTS

### circIQCH is upregulated in primary and metastatic breast cancer

**Figure 1:**
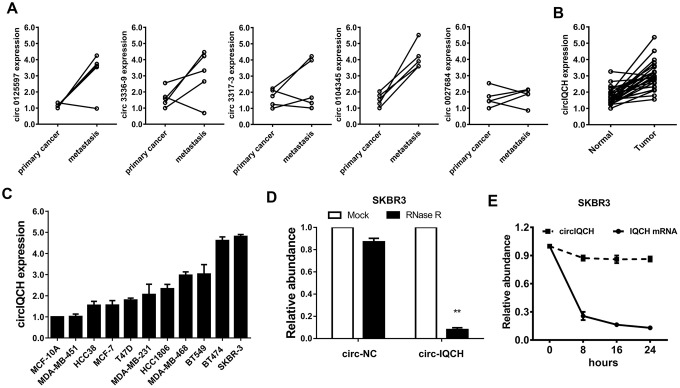
**circIQCH is upregulated in primary and metastatic breast cancer.** (**A**) The expression level of five candidate circRNAs was validated in five paired primary and lung metastatic breast cancer tissues. (**B**) The relative expression of circIQCH in breast cancer tissues and adjacent normal tissues. (**C**) The relative expression of circIQCH in breast cancer cell lines. (**D**) RNase R assay confirmed the circular structure of circIQCH in SKBR3 cell line. (**E**) Circular transcripts of IQCH (circIQCH) was more stable than linear transcripts determined by Actinomycin D treated assay in SKBR3 cell line.

We analyzed the circRNA microarray profiling in our previous study and chose the top five upregulated circRNAs for further validation [22]. Hsa_circ_0104345 was the most upregulated circRNA detected in five paired primary breast cancer tissues and matched metastatic tissues by qRT-PCR analysis (Figure 1A). According to the circBase database and University of California Santa Cruz Genome Browser, we found that hsa_circ_0104345 is derived from gene IQCH (chr15: 67636400-67665771) which is located on chromosome 15p23. Thus, we named hsa_circ_0104345 as circIQCH. We evaluated the expression of circIQCH in breast cancer and adjacent normal tissue by qRT-PCR analysis. The result showed that circIQCH was upregulated in breast cancer tissues (Figure 1B). We found that the expression level of circIQCH was upregulated in breast cancer cell lines compared to normal mammary cell, especially in SKBR3 and BT474 (Figure 1C). Subsequently, RNase R digestion experiment was conducted to verify the circular characteristics of circIQCH (Figure 1D). In addition, Actinomycin D assays revealed that the circular transcript circIQCH is more stable than the linear transcript IQCH in SKBR3 cells (Figure 1E).

### Downregulation of circIQCH suppresses the proliferation of breast cancer cells

**Figure 2:**
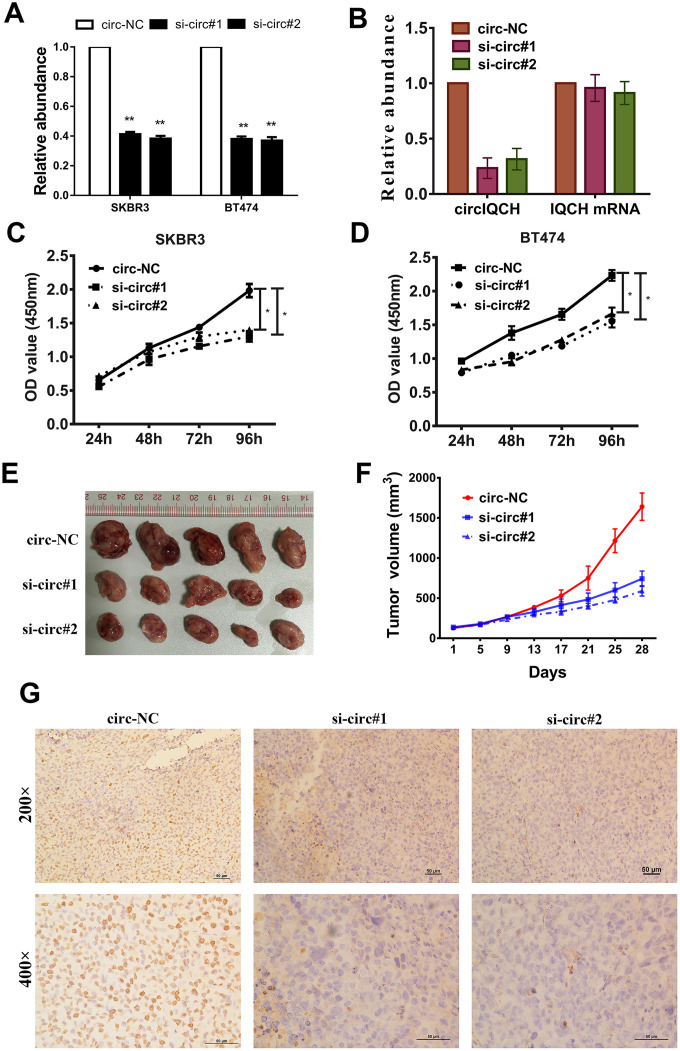
**Downregulation of circIQCH suppresses the proliferation of breast cancer cells.** (**A**) Knock down of circIQCH was assessed by qRT-PCR analysis. (**B**) si-circIQCH decreased the expression of circIQCH while had no effect on linear IQCH mRNA. (**C**, **D**) CCK-8 assays detected cell proliferation. (**E**) Mouse xenograft models were established. (**F**) Tumor volume was estimated in every four days. (**G**) The xenograft tumors were analyzed by immunohistochemistry analysis, and the representative images of ki-67 expression are presented. ^*^P<0.05; ^**^P<0.01.

To investigate whether circIQCH was involved in the proliferation of breast cancer, we next performed loss-of-function assays. Two siRNAs were designed and to knock down circIQCH by targeting the back-splicing region. The expression of circIQCH was decreased after transfected by siRNAs which had no influence on the linear IQCH mRNA expression detected by qRT-PCR analysis (Figure 2A, 2B). CCK-8 assays revealed that circIQCH downregulation suppressed cell proliferation (Figure 2C, 2D). To further assess the functions of circIQCH *in vivo*, mouse xenograft models were established. Consistent with the results in cell experiments, inhibition of circIQCH could reduce the tumor volume (Figure 2E, 2F). In addition, the expression of Ki67 were significantly reduced in tumor tissues of the si-circIQCH groups (Figure 2G).

### Downregulation of circIQCH inhibits the metastasis of breast cancer cells

**Figure 3:**
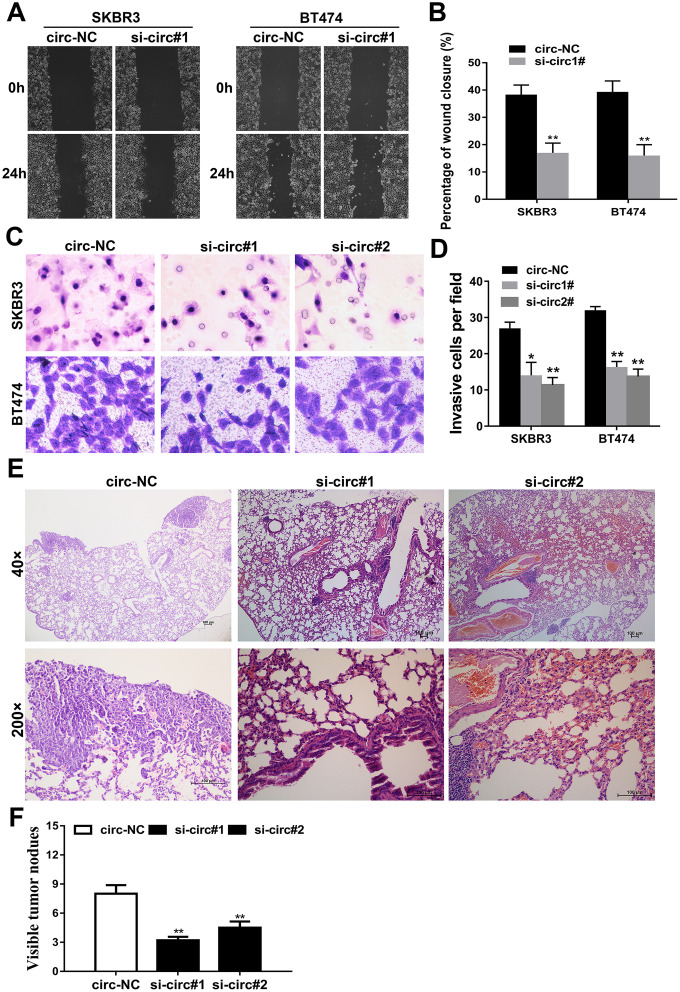
**Downregulation of circIQCH inhibits the metastasis of breast cancer cells.** (**A**, **B**) Wound-healing assays assess the impact of circIQCH on cell migration ability. (**C**, **D**) Transwell assays to evaluate cell migration capability. (**E**) HE-stained sections of lung metastases. (**F**) The number of metastases was counted and recorded.

Wound healing assays and transwell assays were carried out to evaluate the influence of circIQCH on the metastasis of breast cancer. The results showed that silencing the expression of circIQCH could inhibit the percentage of wound closure and migration ability of SKBR3 and BT474 cells (Figure 3A–3D). Additionally, this result could also be observed in lung metastasis experiment *in vivo*. Inhibition of circIQCH could reduce the number of lung metastases, indicating that circIQCH plays an important role in the metastasis of breast cancer (Figure 3E, 3F).

### circIQCH functions as a sponge for miR-145

**Figure 4:**
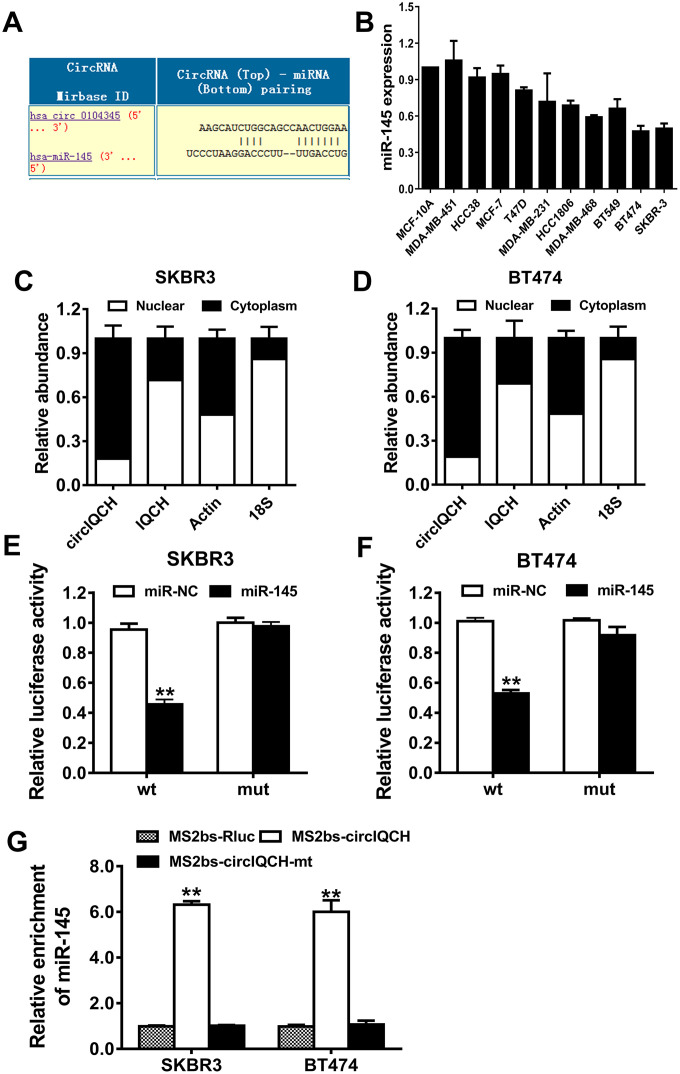
**circIQCH functions as a sponge for miR-145.** (**A**) Predicted binding sites of miR-145 within the circIQCH. (**B**) The relative expression level of miR-145 in breast cancer cell lines. (**C**, **D**) U6, GAPDH and circIQCH in nuclear and cytoplasmic fractions analyzed by qRT-PCR. (**E**, **F**) Luciferase reporter assay of SKBR3 and BT474 cells co-transfected with miR-145 mimics and circIQCH wild type or mutant luciferase reporter. The putative miRNA binding site of circIQCH was mutated. (**G**) MS2-based RIP assay transfected with MS2bs-circIQCH, MS2bs-circIQCH-mt or control. ^*^P<0.05; ^**^P<0.01.

To explore the underlying molecular mechanism of circIQCH in promoting breast cancer progression, we used Circular RNA Interactome to predict the potential circRNA/miRNA interaction (https://circinteractome.nia.nih.gov). Among these candidates, miR-145 was predicted to have the potential to interact with circIQCH (Figure 4A). According to the previously reported studies, miR-145 is downregulated and inhibits tumor progression in breast cancer [23–26]. miR-145 was downregulated in breast cancer cell lines (Figure 4B). circIQCH predominantly existed in the cytoplasm indicating that it could interact with miRNA which is mostly located in the cytoplasm (Figure 4C, 2D). Luciferase reporter assay showed that the luciferase activity decreased after transfected with wild type reporter and miR-145 mimics (Figure 4E, 3F). In order to confirm the direct binding between circIQCH and miR-145, we conducted RNA immunoprecipitation (RIP) assays and the results revealed that miR-145 was primarily enriched in the MS2bs-circIQCH group (Figure 4G). These results indicated that circIQCH functions as a sponge for miR-145 and promotes breast cancer progression.

### circIQCH promotes breast cancer progression via circIQCH-miR-145-DNMT3A axis

**Figure 5:**
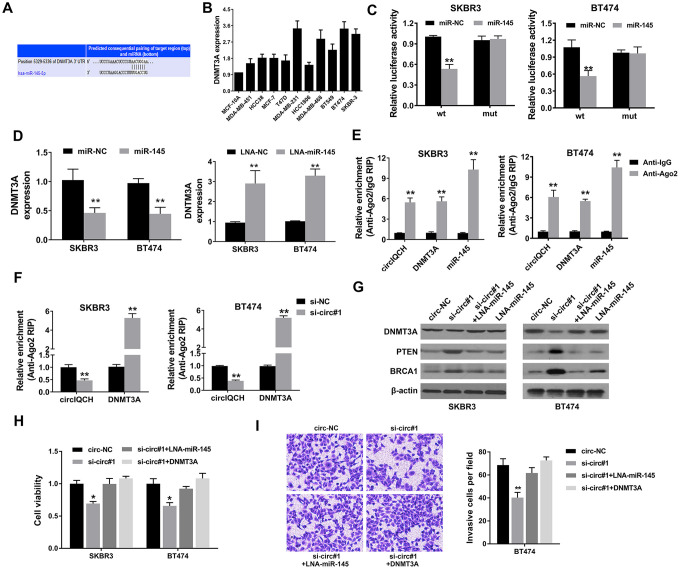
**circIQCH promotes breast cancer progression via circIQCH-miR-145-DNMT3A axis.** (**A**) Predicted binding sites of miR-145 within the 3’-UTR of DNMT3A mRNA according to TargetScan. (**B**) The relative expression level of DNMT3A in breast cancer cell lines. (**C**) Luciferase reporter assay of SKBR3 and BT474 cells co-transfected with miR-145 mimics and the 3’-UTR of DNMT3A wild type or mutant luciferase reporter. The putative miRNA binding site of 3’-UTR of DNMT3A was mutated. (**D**) Expression of DNMT3A was decreased after transfection with miR-145 mimics. Expression of DNMT3A was increased after transfection with miR-145 inhibitors. (**E**) Enrichment of circIQCH, DNMT3A and miR-145 on Ago2 assessed by RIP assay. (**F**) Enrichment of Ago2 to circIQCH was decreased while DNMT3A was increased after knockdown of circIQCH. (**G**) Knockdown of circIQCH resulted in the reduction of DNMT3A expression which was reversed by miR-145 inhibitors. PTEN and BRCA1 was upregulated after silencing circIQCH. (**H**) Cell proliferation rate was detected by CCK-8 assay after exogenously expressing DNMT3A or inhibiting miR-145 in circIQCH silencing SKBR3 and BT474 cells. (**I**) Cell migration ability was validated by transwell assay after exogenously expressing DNMT3A or inhibiting miR-145 in circIQCH silencing SKBR3 cells. ^*^P<0.05; ^**^P<0.01.

We used TargetScan algorithm to predict potential downstream targets of miR-145 and DNMT3A was identified as the candidate target oncogene (Figure 5A). DNMT3A encodes a DNA methyltransferase and modify DNA methylation which plays an important role in tumorigenesis and development in multiple cancers, including breast cancer [27–30]. DNMT3A was found overexpressed in breast cancer cell lines (Figure 5B). Subsequently, luciferase reporter assays and RNA immunoprecipitation assays were conducted to determine whether miR-145 could directly bind the 3’-UTR of DNMT3A mRNA. Luciferase reporter assay showed that the luciferase activity decreased after transfection with miR-145 mimics and wild type 3’-UTR-DNMT3A reporter in SKBR3 and BT474 cells (Figure 5C). Moreover, the expression level of DNMT3A was decreased by miR-145 mimics and increased by miR-145 inhibitors, indicating that DNMT3A is regulated by miR-145 (Figure 5D). Additionally, Ago2 related RIP assays revealed that circIQCH, DNMT3A and miR-145 were all enriched to Ago2 in SKBR3 and BT474 cells (Figure 5E). Downregulation of circIQCH could remarkably increase DNMT3A enrichment to Ago2 (Figure 5F). Western blot analysis revealed that knock down of circIQCH decreased the expression of DNMT3A which could be reversed by the inhibition of miR-145 (Figure 5G). In addition, the expression of two robust tumor suppressors PTEN and BRCA1 was upregulated after silencing circIQCH (Figure 5G). We found that exogenously expressing DNMT3A or inhibiting miR-145 can rescue the effect of si-circIQCH in cell proliferation CCK-8 assay (Figure 5H). Additionally, the migration ability was also reversed in transwell assay after increasing DNMT3A expression or blocking miR-145 in circIQCH knockdown SKBR3 cells (Figure 5I). These results supported that miR-145 and DNMT3A are the downstream effectors of circIQCH in breast cancer.

## DISCUSSION

As a novel type of ncRNA, circRNAs have become a hot topic in the field of life science and attracted the eyes of many researchers. With the huge progress made in high-throughput sequencing technology and bioinformatics algorithms, it is easier for the researchers to detect and characterize thousands of circRNAs [31, 32]. In recent years, an increasing number of circRNAs have been identified and well-studied in the cancer research. A circRNA derived from β-catenin gene promotes hepatocellular carcinoma cell growth through activation of the Wnt pathway by encoding a novel 370-aa β-catenin isoform [33]. circPRKCI is overexpressed and promotes tumorigenesis in lung adenocarcinoma by sponging miR-589 and miR-545 [25]. Circular RNA FLI1, circKIF4A, circRAD18, circ-DNMT1 and circPLK1 were also identified as oncogenic drivers by different mechanisms (maintaining DNA methylation, promoting EMT, reducing apoptosis or activating autophagy) in breast cancer [34–38]. circPRMT5 was discovered as an oncogenic event in urothelial carcinoma of the bladder which can be existed in the exosomes and secreted into serum and urine [39].

In our study, we reanalyzed the circRNA microarray profiling in our previous study and identified hsa_circ_0104345 (circIQCH) as a frequently upregulated novel circRNA in metastatic breast cancer. circIQCH was upregulated in breast cancer tissues, especially in the metastatic sites. We also found that circIQCH was upregulated in breast cancer cell lines. Knockdown of circIQCH inhibited breast cancer cell proliferation and migration to lung in both *in vitro* and *in vivo* assays. In our further study, we performed luciferase reporter assays and RNA immunoprecipitation assays to elucidate the underlying molecular mechanism of circIQCH. We found that circIQCH sponges miR-145 and promotes breast cancer progression by upregulating DNMT3A and downregulating PTEN and BRCA1.

Regarded as the most well-known subclass of non-coding RNA, miRNAs modulate the expression of targeted key genes and intercellular signaling within the tumor microenvironment [40]. miR-145 was predicted and proved as the downstream of circIQCH in our study. Deregulation of miR-145 was found in breast cancer tissues and low expression of miR-145 was correlated with a worse clinical outcome in breast cancer [23–24]. Mucin 1 and ERBB3 were identified as the targets of miR-145 which inhibits migration and proliferation in breast cancer cells [25, 26]. DNMT3A is a DNA methyltransferase which plays an important role in tumorigenesis and metastasis by modifying DNA methylation in multiple cancers, including breast cancer [27–30]. miR-145 regulated the expression level of DNMT3A by binding to the 3’-UTR of DNMT3A mRNA. PTEN and BRCA1 are powerful tumor suppressors in cancer and loss of them will accelerate the process of tumor development [41–42]. According to the published literature, DNMT3A can mediate PTEN and BRCA1 promotor hypermethylation and decrease the expression of PTEN and BRCA1 in several cancers, including breast cancer [43–47]. We found that the expression of PTEN and BRCA1 was increased after silencing circIQCH, indicating that circIQCH might also decrease the expression of these two important tumor suppressors by inducing promoter hypermethylation through circIQCH- miR-145-DNMT3A axis.

In conclusion, our study demonstrated the pivotal role of circIQCH-miR-145-DNMT3A axis in breast cancer growth and metastasis via the mechanism of competing endogenous RNAs. Therefore, circIQCH could be a potential therapeutic target for breast cancer.

## CONCLUSIONS

In conclusion, circIQCH promotes breast cancer growth and metastasis via a novel circIQCH-miR-145-DNMT3A axis and could be a potential therapeutic target for breast cancer.

## MATERIALS AND METHODS

### Patients samples and ethical standards

Fresh primary breast cancer tissues and lung metastatic tissues were collected from the First Affiliated Hospital, University of South China and were frozen in liquid nitrogen immediately after resection. This study was approved by the Ethics Committee of the First Affiliated Hospital, University of South China and performed in accordance with the Declaration of Helsinki. Written informed consent was obtained from all patients before participation in this study. Animal experiment was approved and performed according to the guidelines of Institutional Animal Care and Use Committee of the First Affiliated Hospital.

### Cell culture

All cell lines including MCF-10A, MDA-MB-451, HCC38, BT549, HCC1806, MCF-7, MDA-MB-231, T47D, BT474, SKBR-3 and MDA-MB-468 used in this study were purchased from the American Type Culture Collection (ATCC, USA). Cells were cultured according to the supplier’s instructions and passaged for less than six months. Cell authenticity was verified by DNA fingerprinting.

### Quantitative real-time PCR (qRT-PCR) and transfection

TRIzol (Invitrogen) was used to extract RNA. Isolation of the nuclear and cytoplasmic portions of cellular RNA was performed with NE-PER Nuclear and Cytoplasmic Extraction Reagents (Thermo Scientific). qRT-PCR was performed with SYBR Premix Ex Taq (Takara). Primer information is listed in (Supplementary Tables 1, 2). Transfection was conducted with Lipofectamine 2000 (Invitrogen). The miRNA inhibitors and mimics were purchased from GeneCopoeia (Rockville).

### RNase R digestion assay

After 2 ug extracted total RNA of SKBR3 was incubated with RNase R (3 U/ug) or mock for 30 min at 37°C, the resulting RNA solution was purified and analyzed by qPCR-analysis.

### Actinomycin D assay

SKBR3 breast cancer cells were exposed to 3ug / ml actinomycin D (Sigma) to block the mRNA transcription for 8, 16, and 24 hours. The cells were harvested at certain period and the circIQCH and linear IQCH mRNA were quantified by qPCR-analysis to test the half-life of RNA.

### Cell counting kit-8 (CCK-8) assay

Briefly, 1×10^3^ cells were seeded into a 96-well plate. Ten microliters of CCK-8 solution (Dojindo Laboratories, Japan) was added to each well on a certain day. After incubation at 37 °C for 2h, absorbance at at a wavelength of 450 nM was measured.

### Colony formation assay

A total of 1×10^3^ cells were plated and incubated in each well of a 6-well plate. After incubation at 37°C for 14 days, colonies were fixed with methanol and stained with 0.1% crystal violet. ImageJ software was used to count the colony number.

### Transwell assay and wound healing assay

Transwell assays were conducted using migration chambers (BD Biosciences). Totally, 2×10^4^ cells were added to the upper chambers (serum-free medium) and medium (containing 20% FBS) was added to the lower chambers. Subsequently, cells in the upper chambers were removed, and methanol was used to fix the remaining cells. After staining with crystal violet, the migrated cells were imaged and counted. For the wound healing assay, cells were plated in 6-well plates, and at least three linear wounds were made by scratching with a 200 μL pipette tip. Wounds were imaged at 0 h and 24 h time period.

### Luciferase reporter assay

SKBR3 and BT474 breast cancer cells were seeded into 96-well plates with 5 × 10^3^ cells per well. The putative miRNA binding site of circIQCH and 3’-UTR of DNMT3A was mutated. The constructed reporting vectors (circIQCH-wt/mut or DNMT3A 3’-UTR-wt/mut) and miRNA inhibitors or mimics were cotransfected into cells for 48 hours. Relative luciferase activity was evaluated by the dual-luciferase reporter assay system kit (Promega). All the procedures were conducted according to the manufacturer’s instructions.

### RNA immunoprecipitation (RIP)

Cells were cotransfected with MS2bs-circIQCH, MS2bs-circIQCHmt and MS2bs-Rluc. After 48 h, RIP was conducted with a Magna RIP RNA-Binding Protein Immunoprecipitation Kit (Millipore). The level of miR-145 was quantified after the RNA complexes were purified. The RIP assay for Ago2 was conducted with an anti-Ago2 antibody (Millipore). The abundance of circIQCH, DNMT3A and miR-145 was determined after purification.

### Western blot analysis

Total protein was extracted from RIPA lysis. Then, protein was separated by SDS-PAGE and transferred to PVDF membranes (Millipore). Primary antibody anti-DNMT3A (1:1000, Abcam, USA), anti-PTEN antibody (1:1000, Affinity, USA), anti-BRCA1 antibody (1:1000, CST, USA) and anti-β-actin antibody (1:1000, Affinity, USA) are used to detect certain protein.

### Mouse xenograft model

Cells (1×10^7^) were subcutaneously inoculated into the dorsal flanks of BALB/c nude mice (five mice per group, 4-week-old, female) and treated with an intratumoral injection (40 μL si-NC, si-circIQCH#1 or si-circIQCH#2) every 4 days. We estimated the volume of tumors every four days by formula 0.5×length×width^2^. After four weeks, mice were euthanized, and tumors were weighed. For lung metastasis, cells (1 × 10^5^) were injected through tail veins (five mice per group). The lungs were excised 8 weeks later and the number of metastatic nodules were counted and validated via microscopy of hematoxylin and eosin (HE)-stained sections.

### Statistical analysis

All statistical analyses were performed with SPSS 22.0 software (SPSS Inc., Chicago, IL, USA). Quantitative data are presented as the mean ± standard deviation (SD). Groups were compared using t test. Survival analysis was conducted by Kaplan-Meier plots and log-rank tests. *P*<0.05 was considered statistically significant.

## Supplementary Material

Supplementary Tables
