# Supplementary material for: circIQCH sponges miR-145 to promote breast cancer progression by upregulating DNMT3A expression
Source: Aging (Albany NY). 2020 Aug 3;12(15):15532–45. doi: 10.18632/aging.103746 (PMC7467367; doi:10.18632/aging.103746)
Supplement: Supplementary Tables [file aging-12-103746-s001..pdf]

SUPPLEMENTARY TABLES

Supplementary Table 1. Primer sequences for qRT-PCRs used in this study.

Construct	Species	Direction	Sequence (5' - 3')
18S	Human	Forward	AACTGGAATCGCATCAGGAC
		Reverse	AGGAGCTGCTCTGGGTGTAA
circIQCH	Human	Forward	CCTGATGATGAATGGGTGAA
		Reverse	CACGGAAATCGTTGTTGTTG
DNMT3A	Human	Forward	GCTCTAGACGAAAAGGGTTGGACATCAT
		Reverse	GCTCTAGAGCCGAGGGAGTCTCCTTTTA
GAPDH	Human	Forward	GGAGCGAGATCCCTCCAAAAT
		Reverse	GGCTGTTGTCATACTTCTCATGG
β-actin	Human	Forward	AGCGAGCATCCCCCAAAGTT
		Reverse	GGGCACGAAGGCTCATCATT

Supplementary Table 2. The target sequences of siRNAs used in this study.

siRNA	Species	Target sequences
si-NC	Human	UUCUCCGAACGUGUCACGUTT
si-circIQCH#1	Human	ATCCCATCATTAGGGATTTTA
si-circIQCH#2	Human	ATATCCCATCATTAGGGATTT
